# Association between gut microbiota and common overlapping gastrointestinal disorders: a bidirectional two-sample Mendelian randomization study

**DOI:** 10.3389/fmicb.2024.1343564

**Published:** 2024-05-24

**Authors:** Yuhan Huang, Zhen Kang, Yuhan He, Yi Qiu, Yuhui Song, Weiai Liu

**Affiliations:** ^1^The Second Affiliated Hospital of Hunan University of Chinese Medicine, Changsha, Hunan, China; ^2^The Second Clinical College of Hunan University of Chinese Medicine, Changsha, Hunan, China

**Keywords:** Mendelian randomization, gut microbiota, functional gastrointestinal disorders, functional dyspepsia, irritable bowel syndrome, gastroesophageal reflux disease

## Abstract

**Background:**

The main functional gastrointestinal disorders (FGIDs) include functional dyspepsia (FD) and irritable bowel syndrome (IBS), which often present overlapping symptoms with gastroesophageal reflux disease (GERD), posing a challenge for clinical diagnosis and treatment. The gut microbiota is closely associated with FGIDs and GERD, although the causal relationship has not been fully elucidated. Therefore, we aimed to investigate the potential causal relationship using bidirectional two-sample Mendelian randomization (MR) analysis.

**Materials and methods:**

The genetic data of the 211 gut microbiota were obtained from the MiBioGen consortium (*N* = 14,306, from phylum to genus level) and species level of gut microbiota were acquired from the Dutch Microbiome Project (*N* = 7,738). For FD and IBS, we utilized the FinnGen consortium, whereas, for GERD data analysis, we obtained the IEU OpenGWAS project. The inverse-variance weighted (IVW) method was used as the primary method to calculate causal effect values. Sensitivity analyses were also performed to confirm the robustness of the primary findings of the MR analyses. Moreover, a reverse MR analysis was conducted to assess the likelihood of reverse causality.

**Results:**

Combining the results of the preliminary and sensitivity analyses, we identified that 8 gut microbial taxa were associated with FD. Genus *Lachnospiraceae NK4A136 group* (*p* = 3.63 × 10^−3^) and genus *Terrisporobacter* (*p* = 1.13 × 10^−3^) were strongly associated with FD. At the same time, we found that 8 gut microbial taxa were associated with IBS. Family *Prevotellaceae* (*p* = 2.44 × 10^−3^) and species *Clostridium leptum* (*p* = 7.68 × 10^−3^) display a robust correlation with IBS. In addition, 5 gut microbial taxa were associated with GERD using the IVW approach. In the reverse MR analysis, 2 gut microbial taxa were found to be associated with FD, 5 gut microbial taxa were found to be associated with IBS, and 21 gut microbial taxa were found to be associated with GERD.

**Conclusion:**

The study reveals the potential causal effects of specific microbial taxa on FD, IBS, and GERD and may offer novel insights into the diagnosis and treatment of these conditions.

## 1 Introduction

Functional gastrointestinal disorders (FGIDs) encompass a group of recurrent digestive ailments characterized by symptoms such as postprandial fullness and diarrhea, without clearly identifiable organic abnormalities. The typical symptoms of gastroesophageal reflux disease (GERD) include a sensation of gastric burning and the regurgitation of acidic content (Richter and Rubenstein, 2018). Several studies have demonstrated a significant overlap between GERD and FGIDs, particularly in relation to functional dyspepsia (FD) and irritable bowel syndrome (IBS), which are the most common diseases in FGIDs (Kennedy et al., 1998; Jung et al., 2007; Choung et al., 2012; Quigley and Lacy, 2013). FGIDs and GERD are common digestive diseases. The prevalence of FD based on Internet surveys was found to be 7.2%. Internet-based studies estimated the prevalence of IBS to be 4.1% (Sperber et al., 2021). The global prevalence range for GERD is estimated to be between 8 and 33% (El-Serag et al., 2014). Patients suffering from FGIDs and GERD frequently exhibit mental health conditions, primarily anxiety and depression, as a result of their recurrent symptoms. The prevalence of psychological and behavioral disorders is significantly elevated, thereby establishing a vicious cycle (Guillemot et al., 2005). Due to their high incidence and prolonged disease course, often accompanied by psychological comorbidities, certain patients face challenges in terms of diagnosis and treatment. Additionally, they encounter exorbitant medical expenses and experience a diminished quality of life. Consequently, this has emerged as an urgent medical issue that demands immediate attention and concern from the healthcare community.

The trillions of gut microbiota residing within the human body form a vast micro-ecosystem, exerting pivotal influences on host nutrition metabolism, immune system development and maturation, intestinal endocrine functions, nerve signal transduction, pathogen resistance, cellular proliferation, and angiogenesis (Lynch and Pedersen, 2016). So far, increasing data suggest that the gut microbiota can affect the occurrence of many diseases. Gut microbiota has become a significant focus in understanding the occurrence of gastrointestinal diseases. Relevant studies have shown that the pathogenesis of FD, IBS, and GERD is closely related to intestinal microecological changes, while their correlation has been proved without clear evidence. The overlap of FD, IBS, and GERD symptoms can be attributed to a number of common pathological mechanisms in which the brain–intestinal interaction disorder plays an important role (Powell et al., 2017). For instance, intestinal microbial disorders, alterations in mucosal immune function, changes in intestinal signal transduction such as visceral allergy, and disorders of the central nervous system related to intestinal signal transduction and motor function regulation (Drossman and Hasler, 2016). The overlapping symptoms of FD, IBS, and GERD render clinical diagnosis and treatment challenging. To date, no specific medication exists; pharmacological therapy remains primarily symptomatic, with pronounced short-term efficacy and potentially overlooked long-term side effects (Haastrup et al., 2018). The research progress of gut microbiota is expected to bring new understanding for the diagnosis and treatment of FGIDs and GERD.

MR analysis uses genetic variants strongly associated with exposure as instrumental variables (IVs) to infer causal effects between exposure factors and the outcomes of the study. Numerous MR analyses have been extensively used for the analysis of the correlation between gut microbiota and diseases. However, the investigation into the correlation between gut microbiota and FD, IBS, and GERD remains scant. Only the relationship between gut microbiota and IBS has been individually studied, and the causal relationship between the phylum *Actinobacteria*, genus *Eisenbergiella*, and genus *Flavonifractor* and IBS has been elucidated. The aim of the study was to examine the association between gut microbiota, FD, IBS, and GERD using bidirectional two-sample MR analysis. This will enable further investigation into the causal relationship between dysregulation of gut microbiota and the development of FD, IBS, and GERD, enhance our understanding of the precise connection between gut microbiota, FD, IBS, and GERD, and offer potential for future personalized interventions targeting microflora.

## 2 Method

### 2.1 Study design

**Figure 1:**
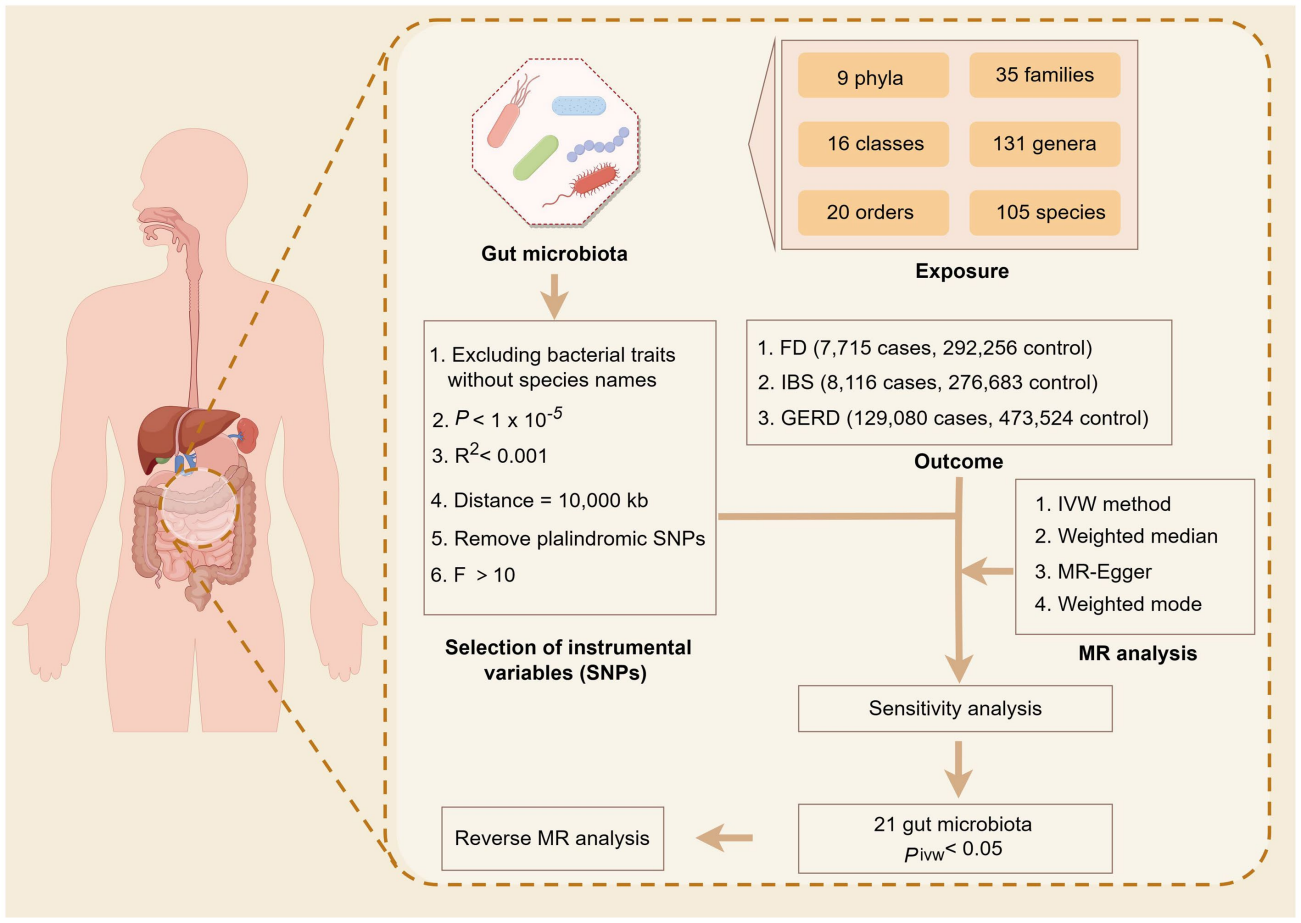
Overview of the Mendelian randomization analysis.

The bidirectional two-sample MR analysis was employed to conduct a comprehensive investigation at the whole gene tissue level, encompassing multiple centers, large sample sizes, and repeated validation. This approach enabled us to assess the relationship between genetic variation and exposure as well as genetic variation and outcome. Subsequently, we inferred the association between exposure and outcome to explore the intricate connection between gut microbiota and common overlapping gastrointestinal diseases. The foundation of this study is based on the three primary hypotheses posited by the MR study (Davies et al., 2018): (1) the correlation hypothesis, where the IVs are strongly correlated with exposure; (2) the exclusivity hypothesis states that IVs can only affect outcomes through exposure and not through any other paths; and (3) the independence hypothesis indicates that IVs are not associated with confounding factors. The research design process is illustrated in Figure 1.

### 2.2 Data sources

The genome-wide association study (GWAS) data are publicly available, ethically approved by the relevant institutions, and can be downloaded. Both the exposed and control groups belong to European populations, which aligns with the Hardy–Weinberg law. The details of the GWAS data in this study are shown in Table 1. The website for relevant data sources and assistive tools is provided in Supplementary material S1.

**Table 1:** Basic information of the GWAS datasets involved in the study.

	Traits	PMID/GWAS ID	Consortium	Population	Sample size	Case/Control
Exposure	Phylum to genus of gut microbiota	33462485	MiBioGen	European	14,306	-
	Species level of gut microbiota	35115690	Dutch Microbiome Project	European	7,738	-
Outcome	FD	-	FinnGen (R8)	European	299,971	7,715/292,256
	IBS	-	FinnGen (R8)	European	284,799	8,116/276,683
	GERD	ebi-a GCST90000514	IEU OpenGWAS	European	602,604	129,080/473,524

#### 2.2.1 Human gut microbiota

The GWAS statistics of gut microbiota from phylum to genus were selected from the MiBioGen. A total of 211 bacterial traits (classified into specific 9 phyla, 16 classes, 20 orders, 35 families, and 131 genera) were obtained with a sample size of 14,306 participants (Kurilshikov et al., 2021). The summary statistics on the species level of gut microbiota were acquired from the Dutch Microbiome Project, which included 105 species and 7,738 participants of European ancestry. Relevant details about the species level of gut microbiota were reported in the original study (Lopera-Maya et al., 2022).

#### 2.2.2 FD, IBS, and GERD

For FD, we utilized the FinnGen R8 dataset. The analysis encompassed 7,715 cases and 292,256 control cases with a total of 20,168,139 SNPs. Regarding IBS, we employed data from 8,116 cases and 276,683 control cases with a total of 20,167,712 SNPs. As for the GERD data analysis, we obtained the data from 129,080 cases from the IEU OpenGWAS and 473,524 control cases, covering a total of 2,320,781 SNPs.

### 2.3 Instrumental variable selection

The gut microbiota at the phyla to genus level includes 297 taxa, comprising 9 phyla, 16 classes, 20 orders, 32 families, 119 genera, and 105 species. To ensure specificity, we excluded species that lacked specific species names (15 in total). Additionally, we excluded four species from the analysis due to having fewer than three available SNPs at the species level of gut microbiota. The outcome and exposed SNPs were subjected to quality checks to ensure the robustness of the data and accuracy of the results: First, the SNPs associated with exposure and outcome have reached the genome-wide significance threshold (*p* < 5 × 10^−8^). However, due to the limited number of satisfying IVs (*p* < 5 × 10^−8^), a more comprehensive threshold has been chosen. Therefore, we have set the significance threshold for gut microbiota as (*p* < 1 × 10^−5^). The GWAS summary data of bacterial taxa from the MiBioGen consortium at *p* < 1 × 10^−5^ can be found in Supplementary Table S1, and the GWAS summary data of species level from the Dutch Microbiome Project can be found in Supplementary Table S2 at a significance level of *p* < 1 × 10^−5^. The outcome data were filtered for SNPs significantly associated with exposure (*p* < 5 × 10^−5^) to obtain more comprehensive results. Second, in order to satisfy the MR hypothesis, the presence of strong linkage disequilibrium (LD) may introduce bias in the results. Therefore, we performed an LD analysis (*r*^2^ < 0.001, distance = 10,000 kb) based on data from the 1,000 Genomes Project and employed PhenoScanner to exclude alleles that directly influence the outcome. Third, the removal of palindromic SNPs is implemented to mitigate the influence of alleles on the outcomes of causal relationships between gut microbiota taxa and FD, IBS, and GERD. The exposures with SNP numbers less than 3 were excluded. The sufficiency of our data precludes the necessity for proxy SNPs. The strength of the exposed genetic instrument is ensured by calculating the F statistic. If F > 10, it is sufficiently strong to avoid weak instrument bias problems in two-sample models.

### 2.4 Statistical analysis

The analysis was conducted using R software (version 4.3.0). The R software package “Two-Sample-MR” was utilized to perform the MR analysis.

### 2.5 MR analysis

First, the inverse-variance weighted (IVW) method was used to evaluate the relationship between gut microbiota and FD, IBS, and GERD. In the absence of horizontal pleiotropy, the IVW method is used as the primary method to calculate causal effect values, using the random-effects model to obtain unbiased estimates. The weighted median (WM), MR-Egger, and weighted mode methods are used as additional methods for the MR analysis. If the number of SNPs with heterogeneity exceeds 50%, the WM result is used as a significant causal effect value, which can reduce the bias of causal effects. Even if up to 50% of SNPs were not valid, the results of MR-Egger would still hold. The weighted mode is used to reduce the error caused by deviations from the estimates of certain genetic variants. FDR was used to correct the *p*-value, when the *p*-value was <0.05, the relationship between gut microbiota and FD, IBS, and GERD was considered to be significant.

### 2.6 Sensitivity analysis

Cochrane’s *Q* test was used to detect heterogeneity, with a significance level of *p*-value <0.05 indicating the presence of significant heterogeneity among the independent variables. The presence of potential pleiotropy in IVs is assessed by evaluating the intercept of MR-Egger regression. In MR-Egger analysis, if the *p*-value of intercept <0.05, it suggests the possibility of horizontal pleiotropy, which may introduce bias to the IVW estimate. To ensure the precision of outcomes pertaining to gut microbiota taxonomic groups with a causal relationship, MR-pleiotropy and MR-PRESSO can automatically identify and remove outliers in IVW linear regression for corrected MR estimates. Additionally, the leave-one-out method was employed to further validate the robustness of the data. The single SNP analysis was utilized to control for potential confounding factors, and the MR Steiger directionality test was conducted to assess whether exposure directly influenced the observed results, thereby enhancing result accuracy.

### 2.7 Reverse MR analysis

In order to investigate the potential causal effects of FD, IBS, and GERD on gut microbiota, we also conducted a reverse MR analysis (using FD, IBS, and GERD as exposures and analyzing gut microbiota as an outcome) to explore the directionality of the causal relationship between gut microbiota and FD, IBS, and GERD. This approach aimed to mitigate the potential impact of reverse causality and enhance the credibility of our research findings. The analytical approach employed in the MR analysis was consistently applied.

## 3 Results

**Figure 2:**
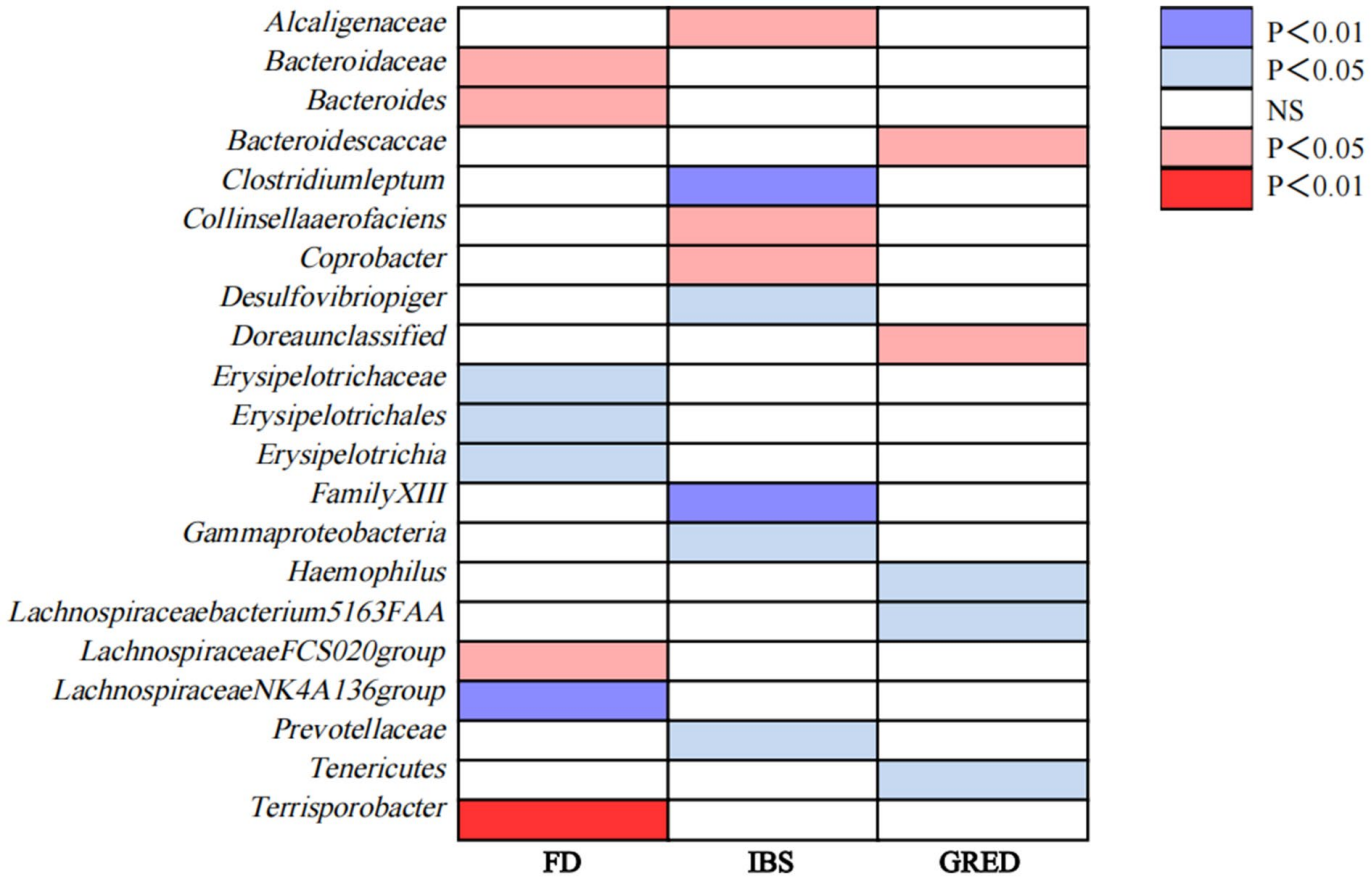
Causal associations of gut microbiota on FD, IBS, and GERD identified at the nominal significance from the IVW method (*p*<0.05/0.01). Red represents the risk bacterial traits for outcomes; blue represents the protective bacterial traits for outcomes, and white represents nocausal bacterial traits for outcomes. NS, no significant association.

**Figure 3:**
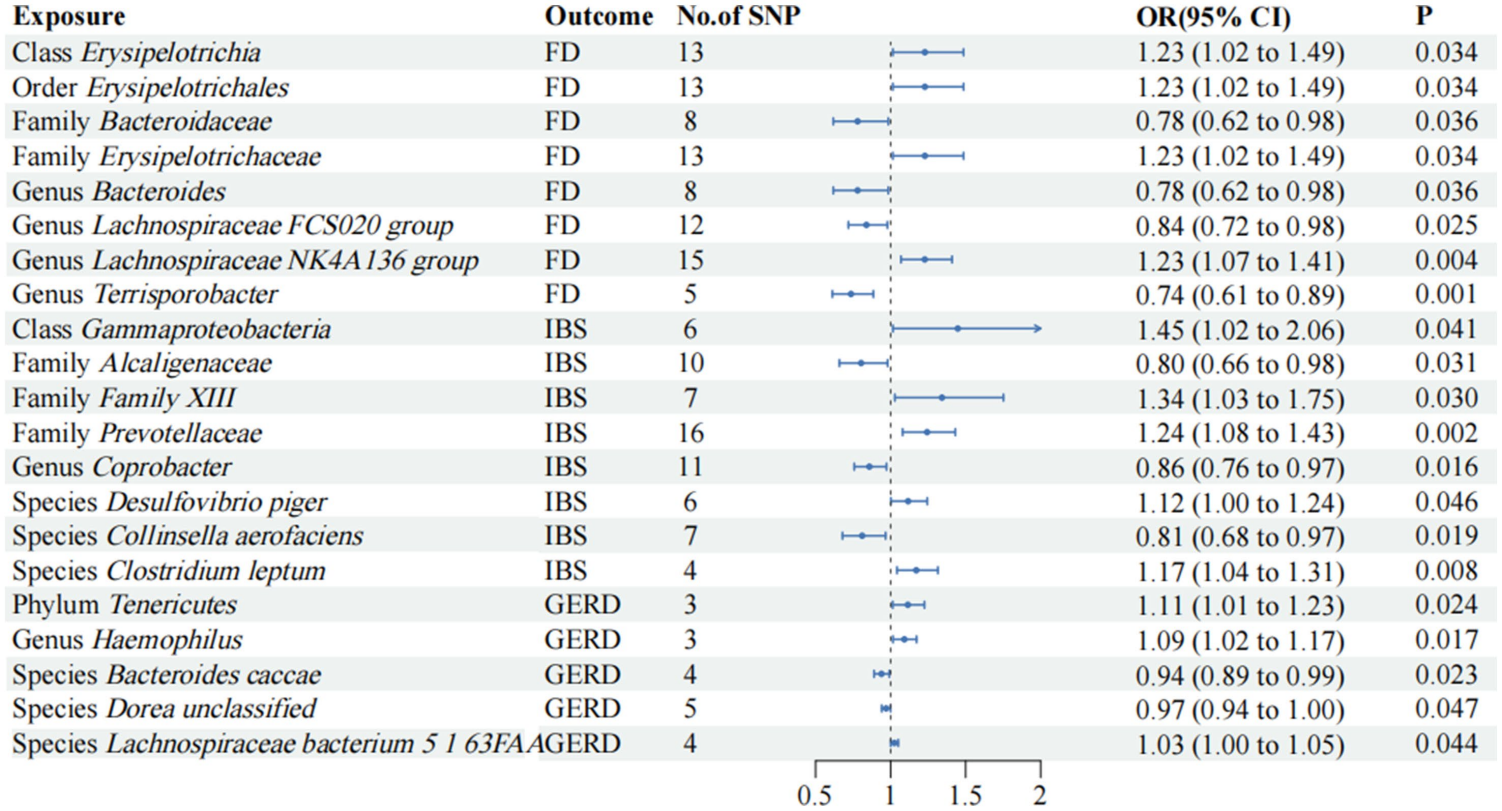
Mendelian randomization analyses of gut microbiota on the risk of FD, IBS, and GERD. Forest plot of IVW analyses. No. of SNP, number of SNPs; OR, odds ratio; CI, confidence interval; *P*, *p*-value of causal estimation in different MR methods.

Even if the results of other methods are not significant, and no pleiotropy and heterogeneity were identified, if the IVW method result is significant (*p* < 0.05), it is regarded as a positive result, provided that the beta values of the other methods are in the same direction. The *F*-statistics of the IVs all exceeded 10, indicating that the estimates are unlikely to be influenced by weak instrumental bias (Supplementary Table S3). The circus plot Figure 2 shows the IVW method results of significant gut microbiota, and the forest plot illustrates the summary causal effects of gut microbiota on the risk of FD, IBS, and GERD based on the IVW method for the primary analysis (Figure 3). The causal effects of MR analysis between exposures and outcomes are shown in Supplementary Table S4.

### 3.1 FD

According to the IVW method, the order *Erysipelotrichales*, class *Erysipelotrichia*, family *Erysipelotrichaceae* (OR, 1.22; 95% CI, 1.01–1.48; *p* = 3.44 × 10^−2^), and genus *Lachnospiraceae NK4A136 group* (OR, 1.22; 95% CI, 1.06–1.40; *p* = 3.63 × 10^−3^) were correlated with higher FD risk (*p* < 0.05). Family *Bacteroidaceae*, genus *Bacteroides* (OR, 0.77; 95% CI, 0.61–0.98; *p* = 3.63 × 10^−2^), genus *Lachnospiraceae FCS020 group* (OR, 0.83; 95% CI, 0.71–0.97; *p* = 2.53 × 10^−2^), and genus *Terrisporobacter* (OR, 0.73; 95% CI, 0.61–0.88; *p* = 1.13 × 10^−3^) were found to be negatively associated with FD risk in the IVW approach (*p* < 0.05).

### 3.2 IBS

We observed positive associations with IBS in the IVW analysis: Class *Gammaproteobacteria* (OR, 1.44; 95% CI, 1.01–2.06; *p* = 4.07 × 10^−2^); family *Family XIII* (OR, 1.34; 95% CI, 1.02–1.75; *p* = 3.03 × 10^−2^); family *Prevotellaceae* (OR, 1.24; 95% CI, 1.07–1.43; *p* = 2.44 × 10^−3^); species *Desulfovibrio piger* (OR, 1.11; 95% CI, 1.00–1.24; *p* = 4.60 × 10^−2^); species *Clostridium leptum* (OR, 1.17; 95% CI, 1.04–1.31; *p* = 7.68 × 10^−3^). Family *Alcaligenaceae* (OR, 0.80; 95% CI, 0.65–0.98; *p* = 3.08 × 10^−2^), genus *Coprobacter* (OR, 0.85; 95% CI, 0.75–0.97; *p* = 1.64 × 10^−2^), and species *Collinsella aerofaciens* (OR, 0.81; 95% CI, 0.67–0.96; *p* = 1.92 × 10^−2^) were negatively associated with IBS risk.

### 3.3 GERD

The IVW analysis revealed significant positive associations with GERD: phylum *Tenericutes* (OR, 1.11; 95% CI, 1.01–1.22; *p* = 2.44 × 10^−2^); genus *Haemophilus* (OR, 1.09; 95% CI, 1.01–1.17; *p* = 1.68 × 10^−2^); and species *Lachnospiraceae bacterium 5 1 63FAA* (OR, 1.02; 95% CI, 1.00–1.05; *p* = 4.37 × 10^−2^). Species *Bacteroides caccae* (OR, 0.93; 95% CI, 0.88–0.99; *p* = 2.27 × 10^−2^) and species *Dorea unclassified* (OR, 0.96; 95% CI, 0.94–0.99; *p* = 4.70 × 10^−2^) were negatively correlated with GERD.

### 3.4 Sensitivity analyses

The causal estimates for amplitude and direction were similar when using the WM, MR-Egger regression, and weighted mode methods. No evidence of pleiotropy at the gut microbiota level was found in the outcomes based on the MR-Egger regression intercept (*p* > 0.05) (Supplementary Table S5). The results from the MR-PRESSO analysis did not indicate any abnormal values (Supplementary Table S6). Furthermore, Cochran’s Q statistic showed no significant heterogeneity (*p* > 0.05) (Supplementary Table S7). Robust results validated using leave-one-out sensitivity tests demonstrated that the exclusion of any single SNP did not impact these findings (Supplementary material S2). The directionality MR Steiger test was passed by all results. The scatter plot can be found in Supplementary material S3.

### 3.5 Reverse MR analysis

**Figure 4:**
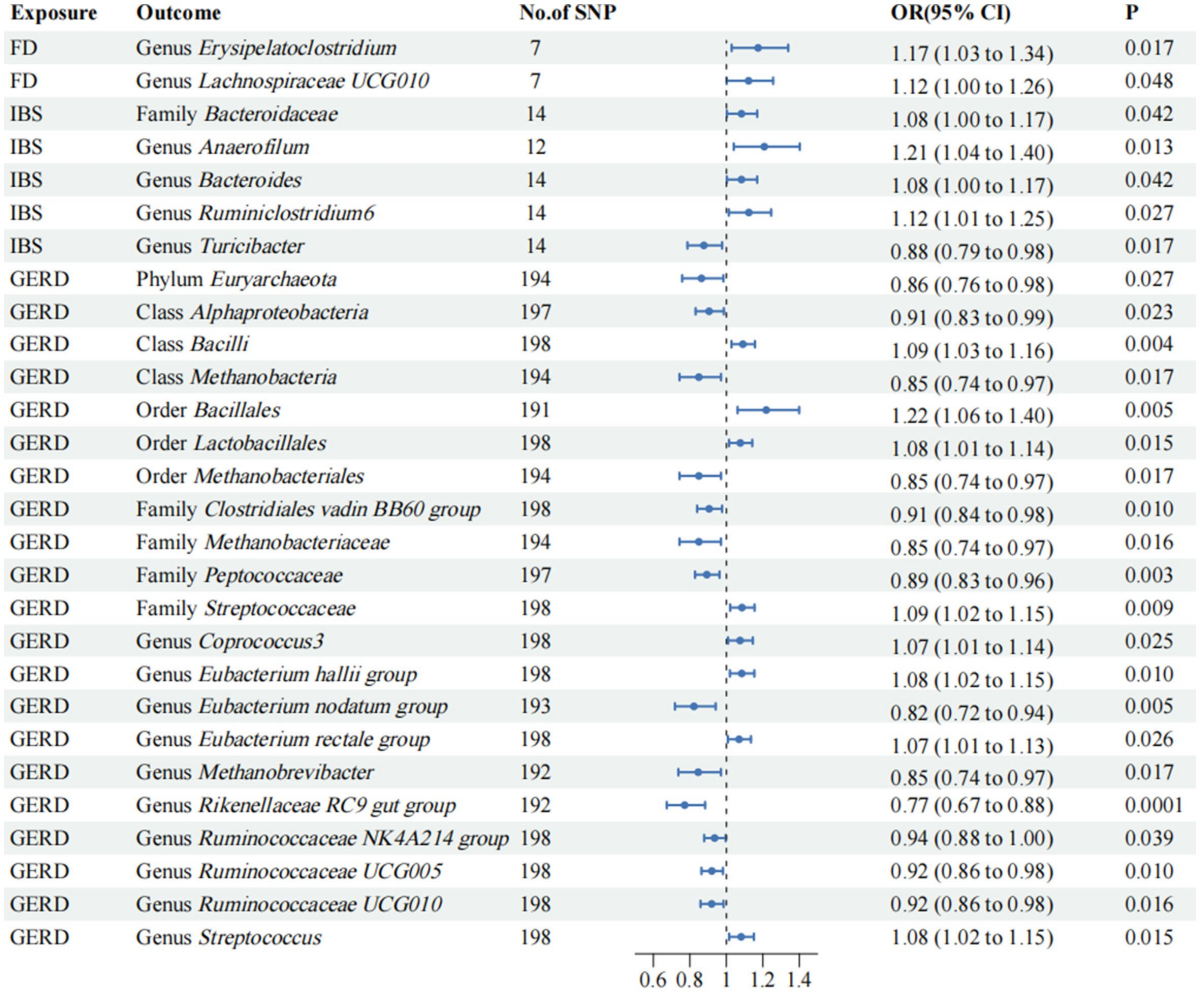
Mendelian randomization analyses of FD, IBS, and GERD on the risk of gut microbiota. Forest plot of IVW analyses. No. of SNP, number of SNPs; OR, odds ratio; CI, confidence interval; *P*, *p*-value of causal estimation in different MR methods.

To evaluate potential reverse causal effects, we conducted a reverse MR analysis utilizing FD, IBS, and GERD as exposures and gut microbiota as the outcome. Consequently, we identified 2 specific gut microbiota significantly associated with FD. Additionally, 5 gut microbiota were found to be linked to IBS while 21 gut microbiota showed an association with GERD; the associations are depicted in Figure 4, respectively. Class *Alphaproteobacteria* may potentially contribute to the observed heterogeneity, but no pleiotropy was detected (*p* > 0.05). Nevertheless, the results lack stability. The reverse MR analysis for the causal effect of outcomes on gut microbiota is provided in Supplementary Table S8.

## 4 Discussion

In recent years, the majority of microbiota research has primarily focused on fecal samples, highlighting an urgent necessity for advancements and breakthroughs in investigating gut microbiota and their associated metabolites. The present study is pioneering in its use of MR analysis to comprehensively elucidate the potential causal relationship between gut microbiota and common overlapping gastrointestinal disorders. The gastrointestinal tract is an integrated structure. Visceral hypersensitivity, dysregulation of the central nervous system, and disturbances in intestinal microecology represent the primary common pathophysiological mechanisms underlying the overlap of FGIDs and GERD symptoms (Ford et al., 2020). Abnormal regulation of the central system is a crucial pathophysiological basis underlying the manifestation of overlapping symptoms. The brain–gut axis (BGA) is a bidirectional connection between the nervous system and the gastrointestinal tract (Zhang et al., 2019). The brain–gut–microbiota axis (BGMA) consists of the BGA, the related endocrine, immune systems, and gut microbiota, which together form a complex feedback network and participate in the integration of gastrointestinal physiological and pathological processes (Dalile et al., 2019). A complex and inseparable inter-regulatory relationship occurs between the gut microbiota and the traditional BGA. The precise role of gut microbiota in FD, IBS, and GERD remains to be thoroughly investigated. Our study is therefore fundamental as an attempt to fill up gaps in our understanding of clear evidence between gut microbiota and FD, IBS, and GERD.

The bacterial load is inversely correlated with the quality of life (Zhong et al., 2017), and recent studies have reported relatively elevated levels of *Proteobacteria*, *Firmicutes*, *Bacteroidota*, *Fusobacteriota*, and *Actinomyces* in the gut microbiota of patients with FD (Paroni Sterbini et al., 2016). The findings of other studies have demonstrated that the level of *Streptococcus* in patients with FD is significantly elevated compared to the healthy control group (Fukui et al., 2020). Additionally, an inverse relationship has been observed between the abundance of *Streptococcus* and *Prevotella*, *Veillonella*, and *Actinomyces* (Zhong et al., 2017). Genus *Bacteroides* is classified within the family *Bacteroidaceae.* It is recognized as an obligate anaerobic, anti-bile bacterium and represents one of the predominant genera in the intestinal tract (Wexler, 2007). Short-chain fatty acids (SCFA) are crucial signaling molecules that play an important role in the BGMA, and they originate from the fermentation of dietary fibers in the intestine by anaerobic bacteria in the colon, mainly *Firmicutes* and *Bacteroidota*. The production of SCFA by Bacteroidetes can influence the BGMA, maintain the integrity of the intestinal barrier, and regulate gastrointestinal motility, thus affecting the development of FD (Fellows et al., 2018). These conclusions are consistent with our results. *Erysipelatoclostridium* is a genus of family *Erysipelotrichaceae*; *Erysipelotrichaceae* is a family of order *Erysipelotrichales*, and class *Erysipelotrichia* within phylum *Firmicutes*. *Firmicutes* can regulate BGMA-mediated FD by producing SCFA. The efficacy of STW 5-II in promoting the enrichment of *Erysipelotrichaceae* and inhibiting the growth of pathogenic species from the family *Enterobacteriaceae* in human stool samples has been demonstrated (Ammar et al., 2023). In reverse MR analyses, FD is also a risk factor for the genus *Erysipelatoclostridium*. Except for our results of this analysis, there is no actual evidence to prove the clear mechanism of order *Erysipelotrichales*, class *Erysipelotrichia*, family *Erysipelotrichaceae*, and FD, and the specific occurrence process needs further study. In line with our analysis findings, a study revealed that FD mice exhibited a significant gut microbiota disorder, particularly within the genus *Lachnospiraceae NK4A136 group* (Zhang et al., 2020). Our results confirm that the genus *Lachnospiraceae NK4A136 group* is a risk factor for FD and is strongly associated with FD (*p* = 3.63 × 10^−3^). The specific mechanisms of the genus *Lachnospiraceae NK4A136 group* and FD deserve further investigation (Chen et al., 2018). Genus *Lachnospiraceae FCS020 group*, a member of the *Lachnospiraceae* family, is capable of producing SCFA such as acetate and butyrate, which possess anti-inflammatory properties and are generally recognized as beneficial bacteria (Sivaprakasam et al., 2016; Sorbara et al., 2020), this is in line with our findings. In addition, the results of our study demonstrated a significant negative correlation between the genus *Terrisporobacter* and the progression of FD (*p* = 1.13 × 10^−3^), which remains unverified by previous studies and thus necessitates further investigation.

Modern medical research has shown that gut microbiota, intestinal permeability, intestinal immunity, intestinal dynamics, visceral sensation, brain–gut interactions, and psychosocial status are important factors in the pathogenesis of IBS. Its pathogenesis is similar to that of FD, and BGMA also plays an important role in the pathogenesis of IBS (Lacy et al., 2021). The findings of our study unravel, for the first time, that some gut microbiota can indeed sway the onset of IBS. It is imperative to note that the species *Clostridium leptum* displays a robust correlation with IBS (*p* = 7.68 × 10^−3^), albeit no definitive evidence is currently available to corroborate its influence on the incidence of IBS. A study has shown that oral administration for IBS treatment can lead to a reduction in the abundance of *Bacteroides Prevotellaceae* (Simpson et al., 2020), thereby suggesting a potential link between variations in *Prevotellaceae* abundance and the occurrence of IBS. Our results first confirmed that *Prevotellaceae* was strongly correlated with IBS (*p* = 2.44 × 10^−3^) and was a risk factor for IBS. A quantitative analysis of the stool samples from patients with IBS revealed a significant reduction of 97% in the abundance of species *Collinsella aerofaciens* within their fecal matter (Malinen et al., 2010). These evidences prove that the family *Prevotellaceae* and species *Collinsella aerofaciens* are related to IBS. The study shows a high relative abundance of the species *Desulfovibrio piger* in the feces of IBS patients (Villanueva-Millan et al., 2024). Our findings suggest that the species *Desulfovibrio piger* is a risk factor for IBS. However, in addition to our research, the specific relationship between class *Gammaproteobacteria*, family *Alcaligenaceae*, family *Family XIII*, genus *Coprobacter*, and IBS remains unclear. Our result further substantiates the impact of these microbial communities on IBS, although a comprehensive understanding of the underlying mechanism still necessitates additional investigation.

The presence of evident abnormalities in both the esophageal and gut microbiota of GERD has been confirmed, with a close association between flora disturbance and the severity of esophagitis as well as heightened visceral sensitivity. This primarily manifests as reduced diversity and uneven distribution of gut microbiota species. Disturbance of intestinal microorganisms is one of the important causes of GERD, and BGMA is closely related to GERD (Liang et al., 2018; Selling et al., 2018; Jang et al., 2020). Research detected a high abundance of *Streptococcus* in the gastrointestinal tract of a rat model exhibiting symptoms of GERD (Zaidi et al., 2016). Interestingly, in the reverse MR analyses, GERD emerged as a risk factor for the genus *Streptococcus* and family *Streptococcaceae*, the exact reasons for which are unclear and necessitate further investigation. Among esophageal microbiota, dysregulated *Haemophilus* abundance is associated with GERD (Di Pilato et al., 2016). However, it was found that the altered immune ecological niche of the esophagus may also be related to the systemic inflammatory effects of the gut microbiota (Münch et al., 2019), and our results confirm that genus *Haemophilus* is a risk factor for GRED. Additionally, our study has provided preliminary evidence, indicating significant positive associations between species *Lachnospiraceae bacterium 5 1 63FAA* and GERD within the species level. Conversely, species *Dorea unclassified* and species *Bacteroides caccae* were found to be negatively correlated with GERD. Our results reveal that the pathogenesis of GERD is closely related to gut microbiota. In clinical practice, modulation of gut microbiota by these microorganisms holds promise for both prevention and treatment of GERD.

Our research has several advantages. (1) All GWAS data were obtained from the European population, effectively avoiding bias due to ethnic differences, thereby enabling a more accurate reflection of the causal relationship between gut microbiota and common overlapping gastrointestinal diseases. (2) This study also provided specific gut microbiota involved in the pathogenesis of FD, IBS, and GERD and added species level. (3) Genetic variation of gut microbiota was obtained from the largest available GWAS metadata, and GWAS data at the species level of gut microbiota were added. (4) To enhance the credibility of our study, we employed multiple MR analysis methods to ensure the accuracy of our results, PhenoScanner was used to remove confounders, FDR was consistently applied for adjustment of *p*-values, and sensitivity analyses using multiple methods did not reveal significant heterogeneity or pleiotropy in the instrumental variables. However, our study has several limitations. First, although our analysis includes a total of 297 microbial groups, we only investigated potential causal relationships with common overlapping gastrointestinal diseases for 101 species. No further exploration was conducted for other microbial groups. Second, the predominantly European ancestry of the study participants may limit the generalizability of the findings to other populations. Third, it was not possible to determine whether there was any overlap in participants between the GWAS conducted for exposure and outcome variables used in the two-sample MR analysis.

## 5 Conclusion

We conducted a comprehensive screening of the gut microbiota associated with FD, IBS, and GERD. We identified 8 gut microbial taxa that were associated with FD. Genus *Lachnospiraceae NK4A136 group* and genus *Terrisporobacter* showed strong association with FD. Additionally, we found that 8 gut microbial taxa were associated with IBS. Family *Prevotellaceae* and species *Clostridium leptum* display a robust correlation with IBS. Furthermore, 5 gut microbial taxa were associated with GERD. In reverse MR analysis, 2 gut microbial taxa were found to be associated with FD, 5 gut microbial taxa were found to be associated with IBS, and 21 gut microbial taxa were found to be associated with GERD. Our findings provide substantial theoretical support for modulating the composition of gut microbiota to enhance therapeutic efficacy and facilitate the development of microbiome-based therapeutic strategies as well as microbial biomarkers for FD, IBS, and GERD. It is worth noting that further observations or laboratory-based studies are imperative to validate these findings.

## Data availability statement

The datasets presented in this study can be found in online repositories. The names of the repository/repositories and accession number(s) can be found in the article/Supplementary material.

## Author contributions

YHu: Writing – original draft. ZK: Data curation, Writing – review & editing. YHe: Investigation, Writing – review & editing. YQ: Investigation, Writing – review & editing. YS: Investigation, Writing – review & editing. WL: Funding acquisition, Resources, Supervision, Writing – review & editing.
