# Supplementary material for: Association between gut microbiota and common overlapping gastrointestinal disorders: a bidirectional two-sample Mendelian randomization study
Source: Front Microbiol. 2024 May 24;15:1343564. doi: 10.3389/fmicb.2024.1343564 (PMC11157101; doi:10.3389/fmicb.2024.1343564)
Supplement: Supplementary file 2 [file Data_Sheet_1.docx]

Supplementary Material 1

211 gut bacteria from MiBioGen (<https://mibiogen.gcc.rug.nl/>)

FinnGen database (<https://www.finngen.fi/en>)

IEU OpenGWAS (<https://gwas.mrcieu.ac.uk/>)

Figdraw for assistance in making Figure 1 ([www.figdraw.com](file:///D:\孟德尔随机化研究\正文\正文\www.figdraw.com))

Chiplot for assistance in making Figure 2 ([ChiPlot](https://www.chiplot.online/))

PhenoScanner to exclude alleles that directly influence the outcome.

([www.phenoscanner.medschl.cam.ac.uk](file:///D:\孟德尔随机化研究\正文\正文\www.phenoscanner.medschl.cam.ac.uk))
