# Supplementary material for: Association between gut microbiota and common overlapping gastrointestinal disorders: a bidirectional two-sample Mendelian randomization study
Source: Front Microbiol. 2024 May 24;15:1343564. doi: 10.3389/fmicb.2024.1343564 (PMC11157101; doi:10.3389/fmicb.2024.1343564)
Supplement: Supplementary file 4 [file Data_Sheet_3.docx]

Supplementary Material 3


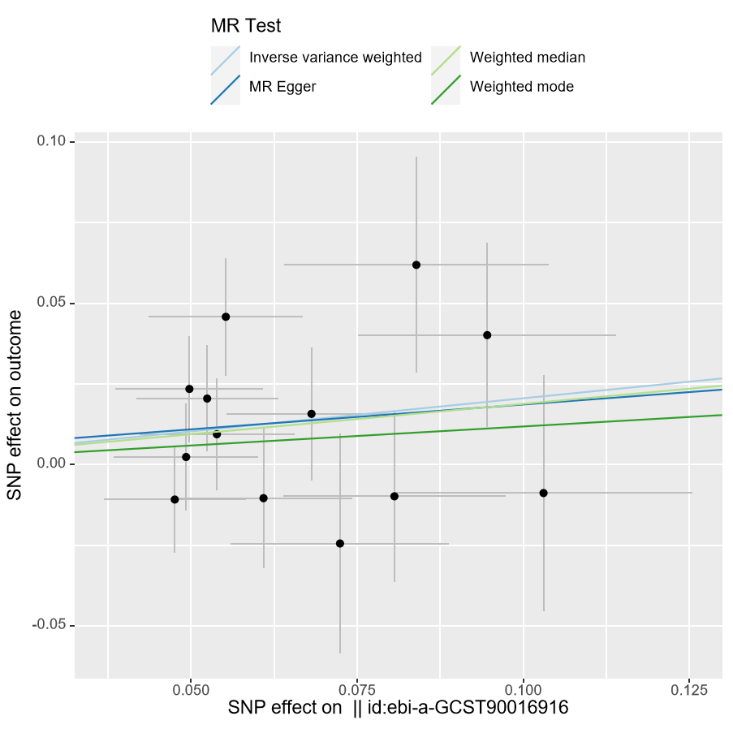

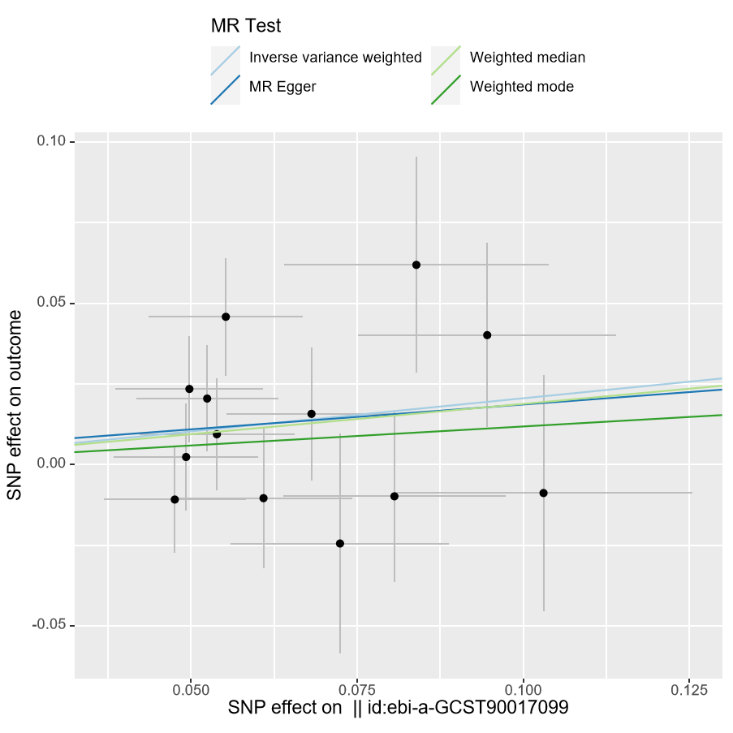


（A）Class *Erysipelotrichia* （B）Order *Erysipelotrichales*


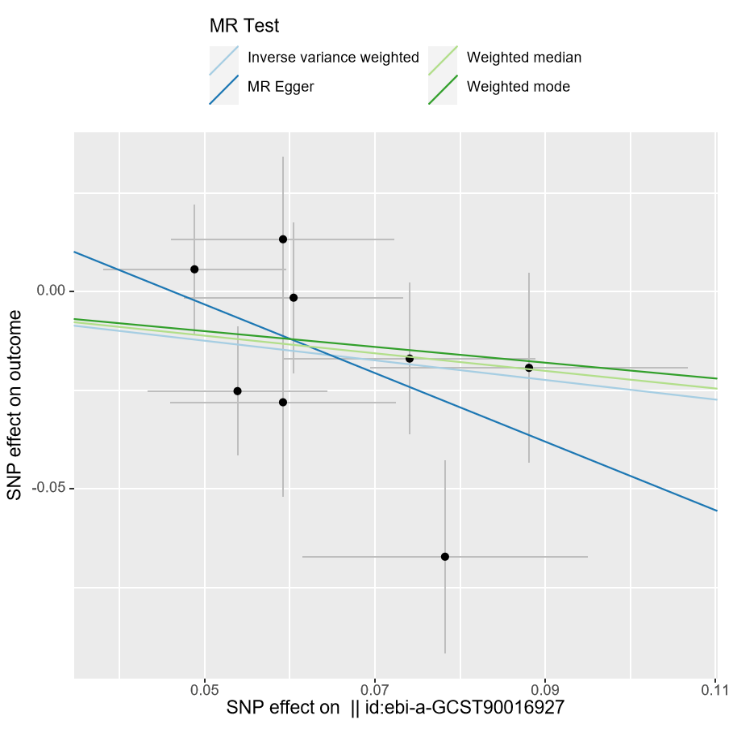

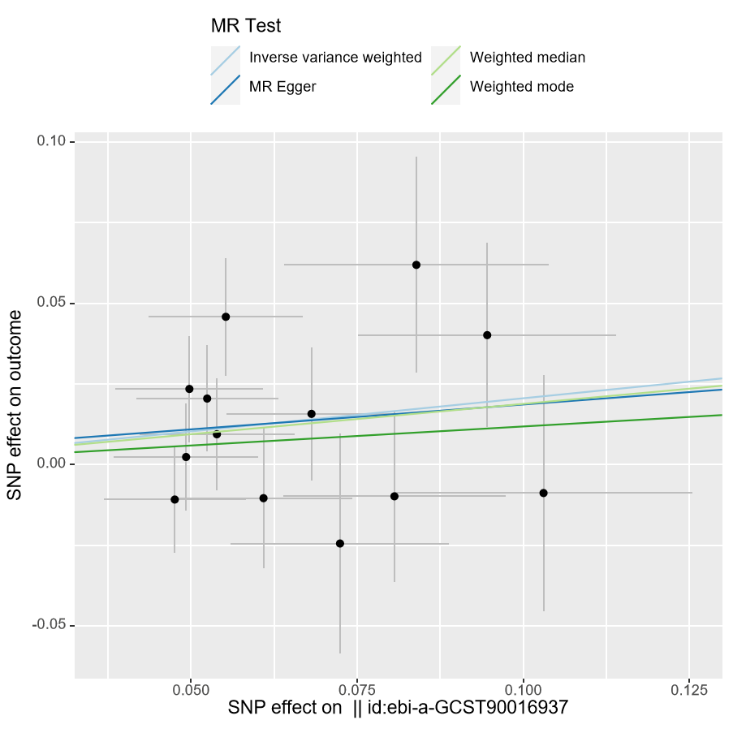


（C）Family *Bacteroidaceae* （D）Family *Erysipelotrichaceae*


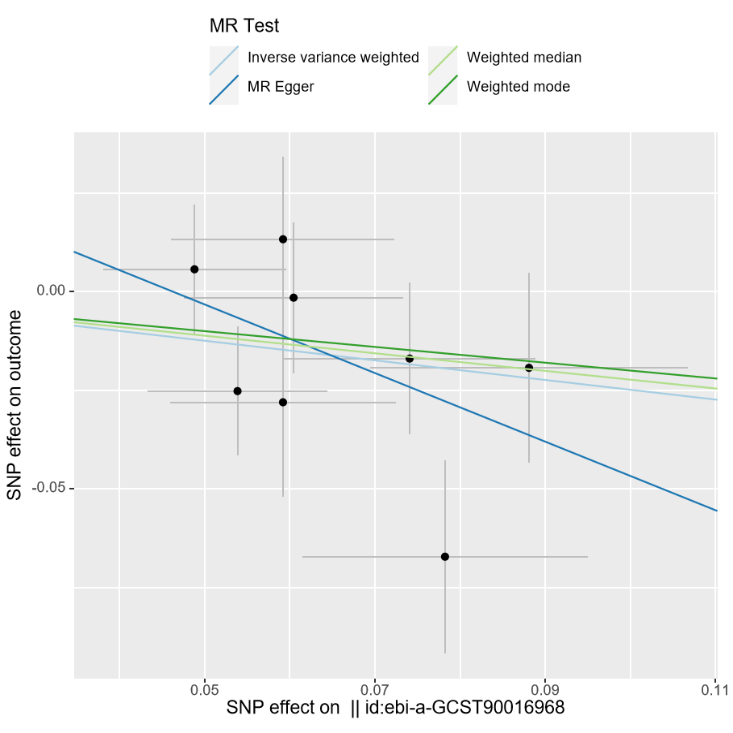

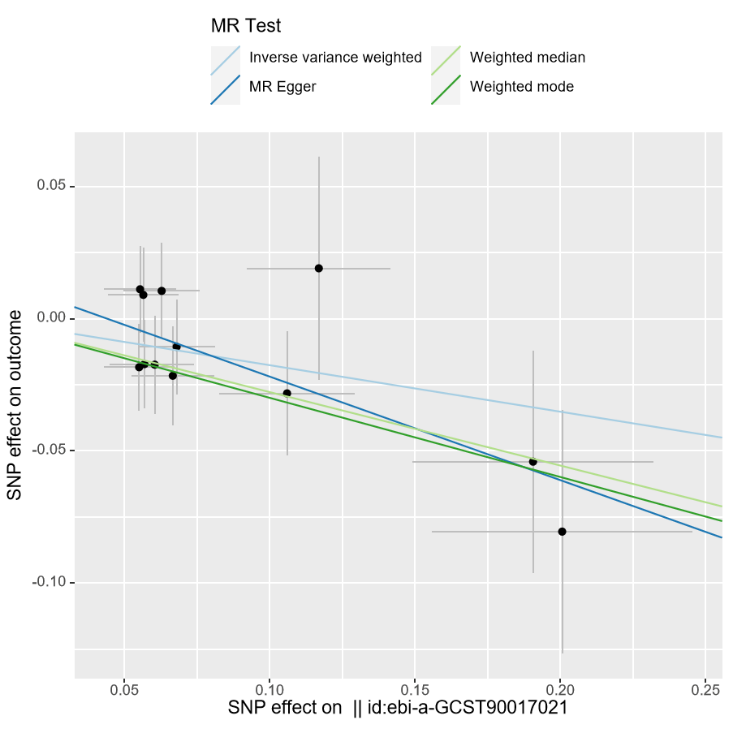


（E）Genus *Bacteroides* （F）Genus *Lachnospiraceae FCS020 group*


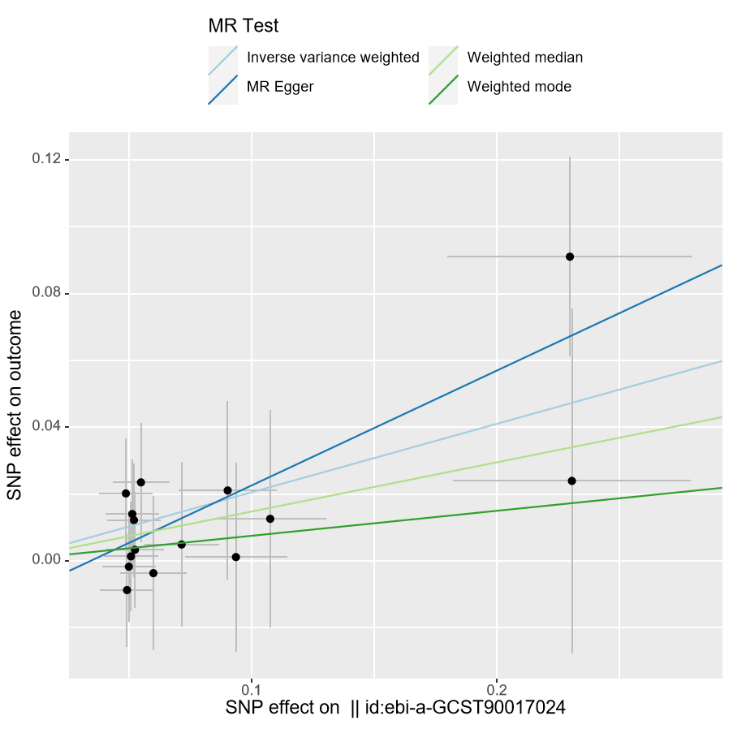

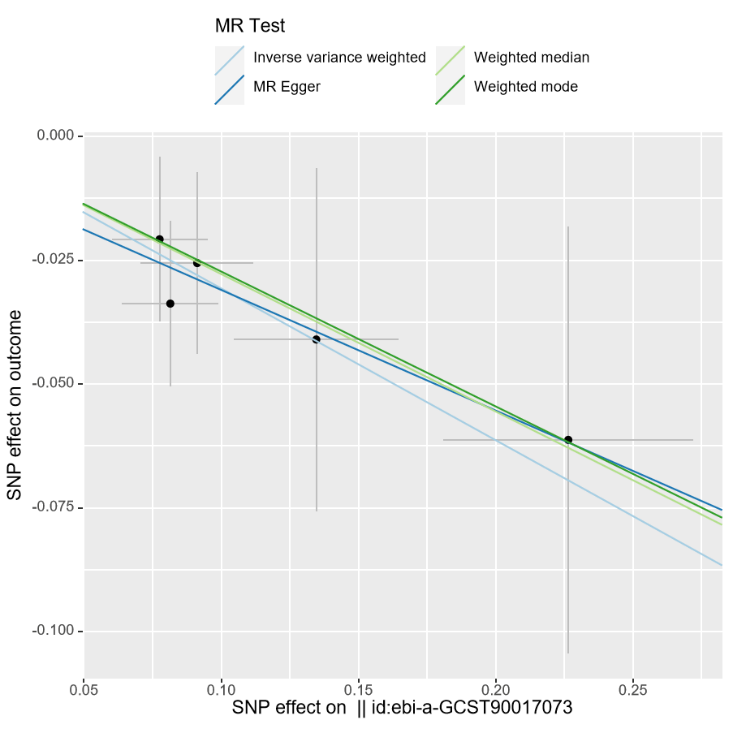


（G）Genus *Lachnospiraceae NK4A136 group* （H）Genus *Terrisporobacter*

**FD**


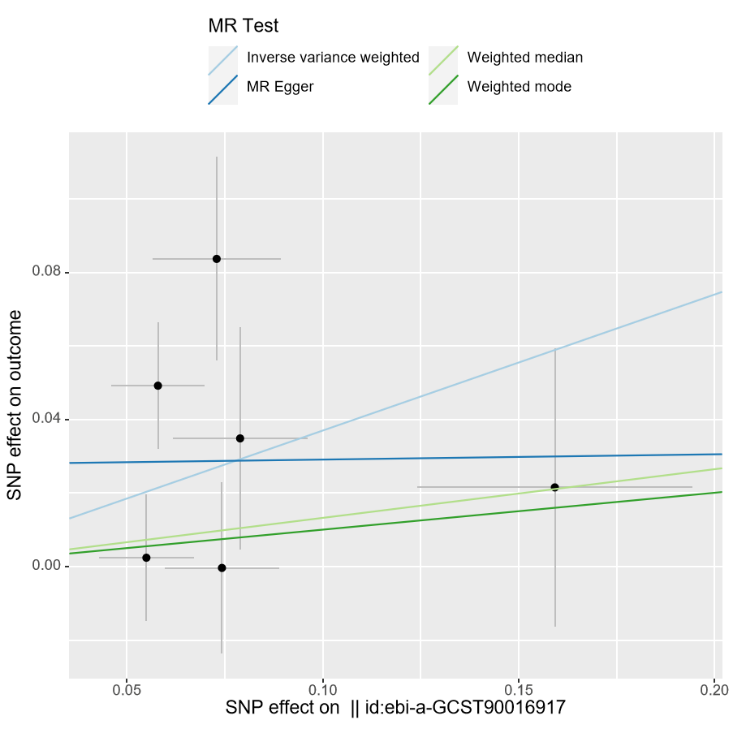

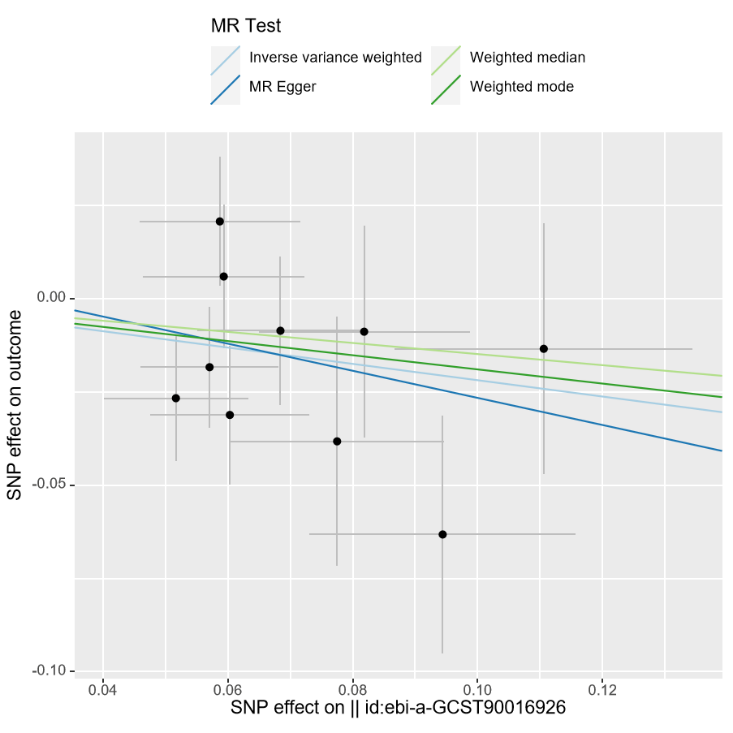


（A）Class *Gammaproteobacteria* （B）Family *Alcaligenaceae*


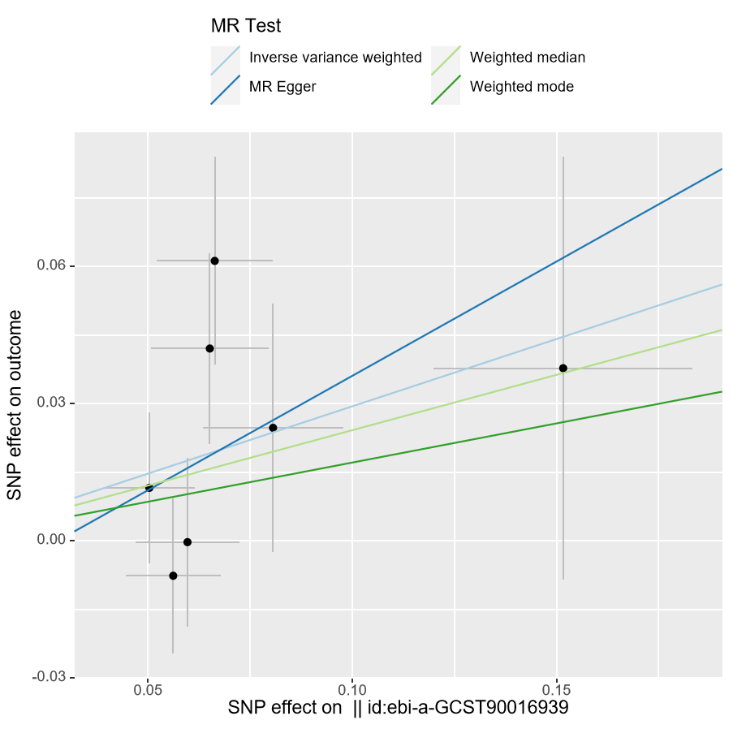

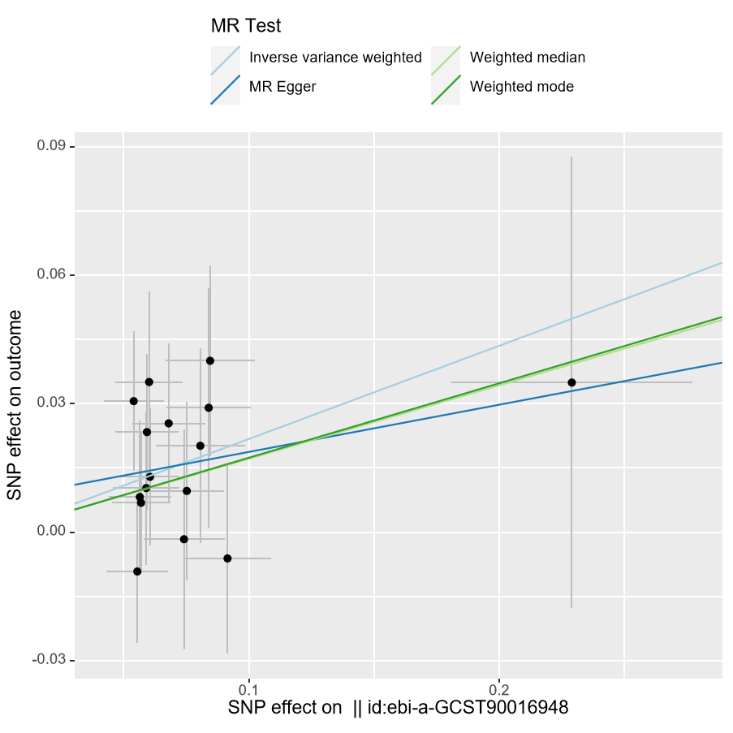


（C）Family *Family XIII* （D）Family *Prevotellaceae*


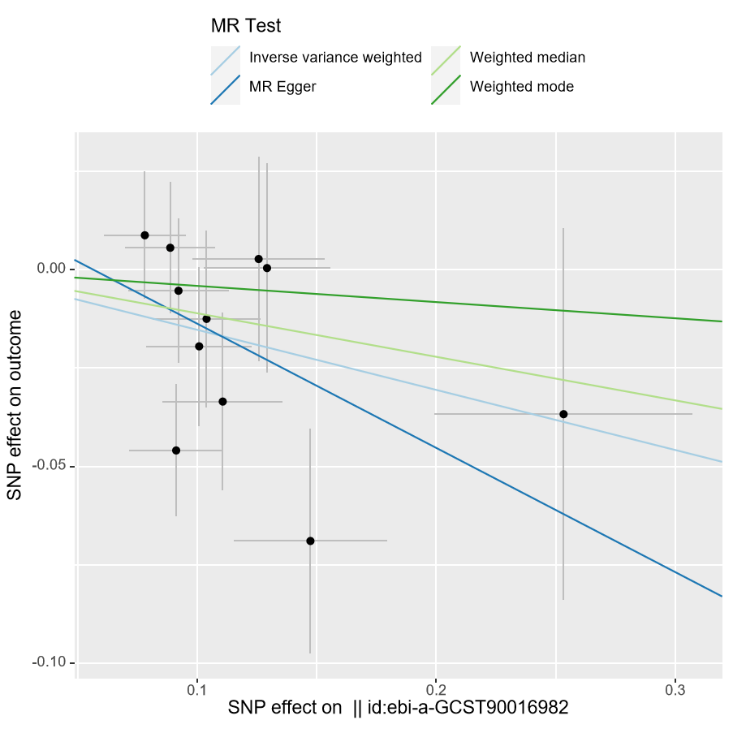

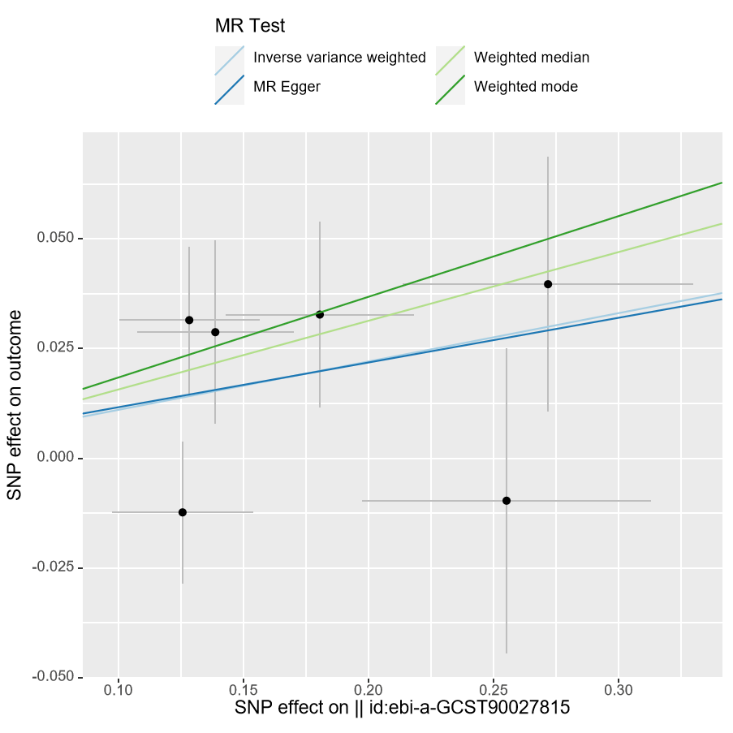


（E）Genus *Coprobacter* （F）Species *Desulfovibrio piger*


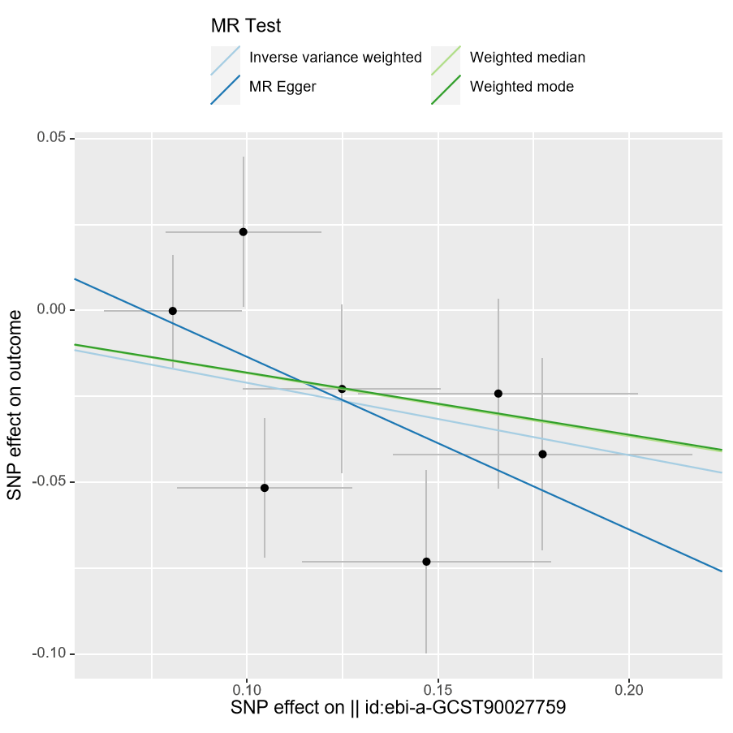

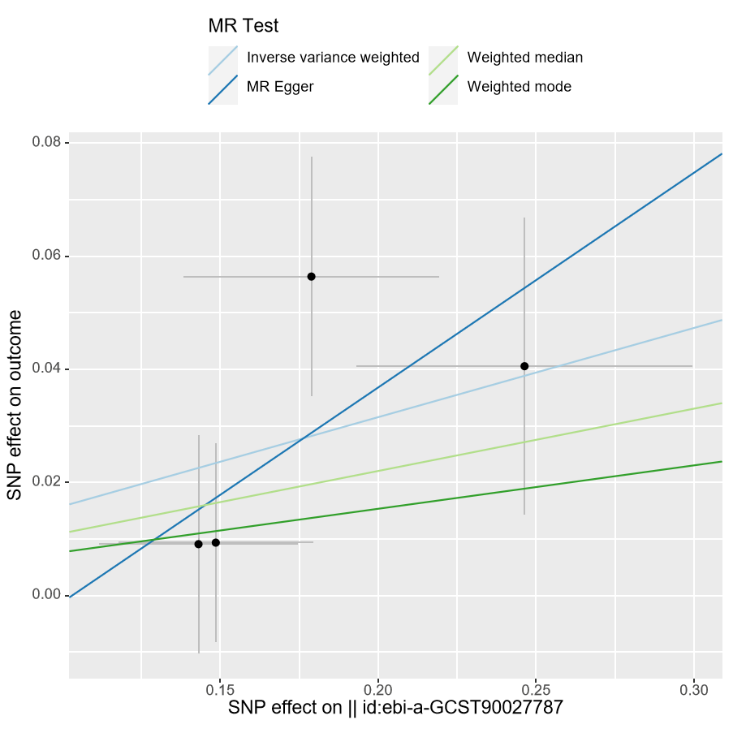


（G）Species *Collinsella aerofaciens* （H）Species *Clostridium leptum*

**IBS**


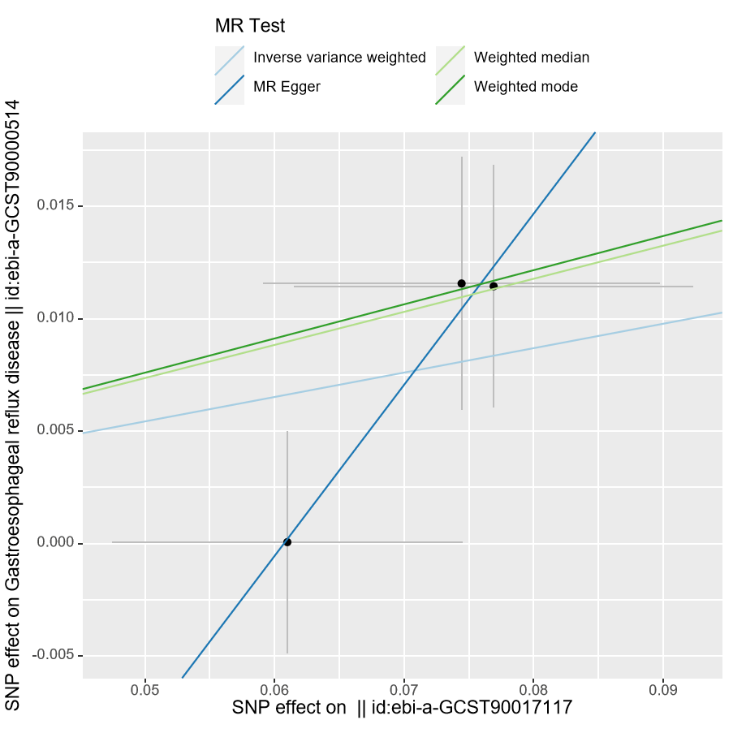

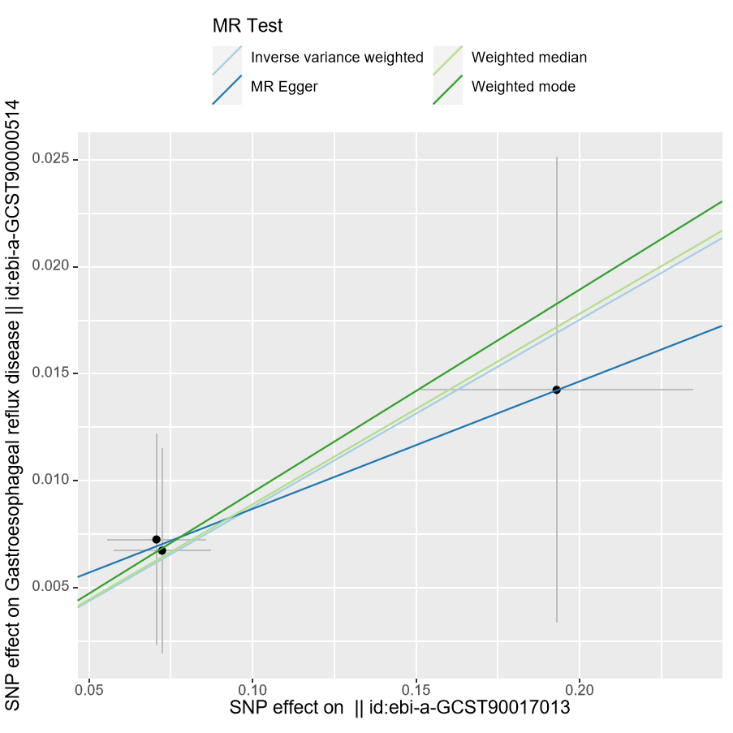


（A）Phylum *Tenericutes* （B）Genus *Haemophilus*


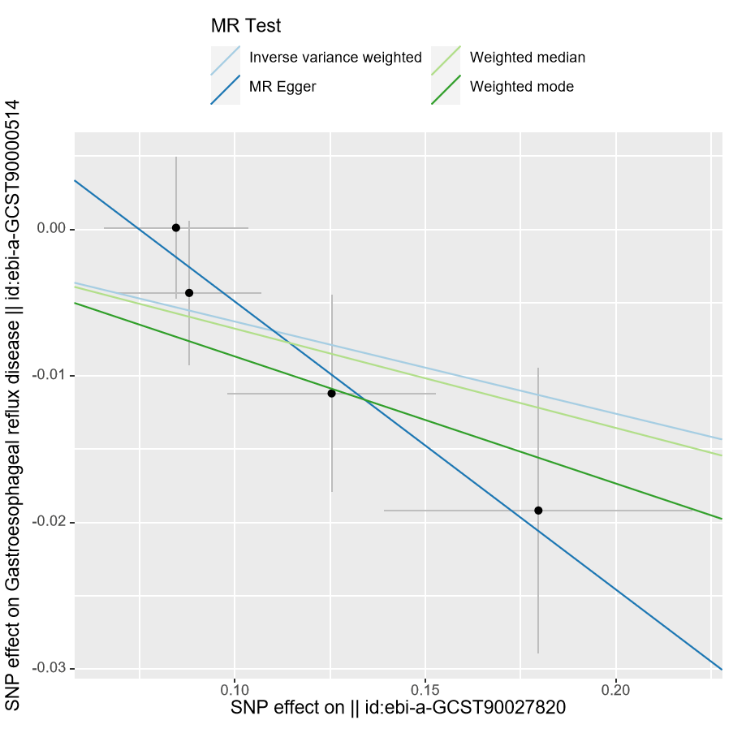

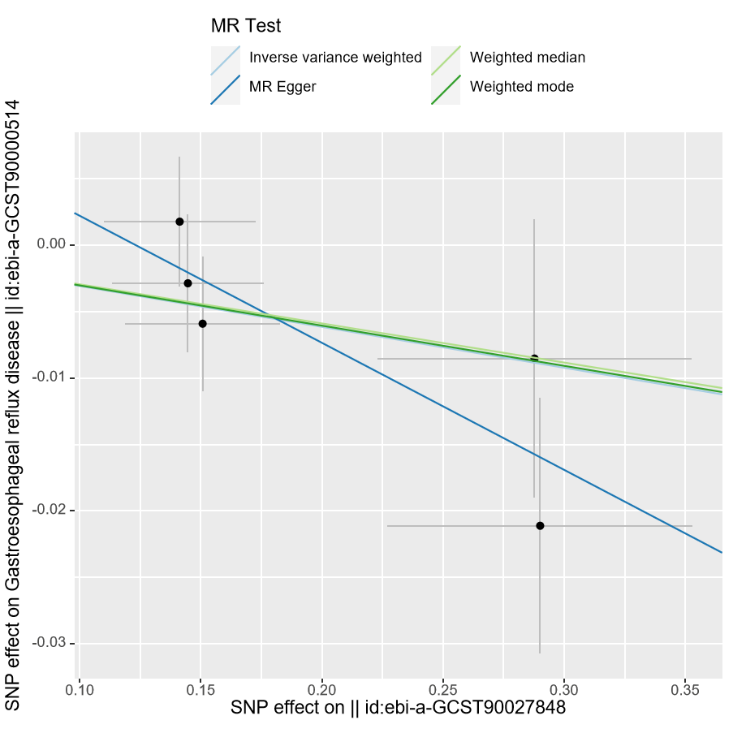


（C）Species *Bacteroides caccae* （D）Species *Dorea unclassified*


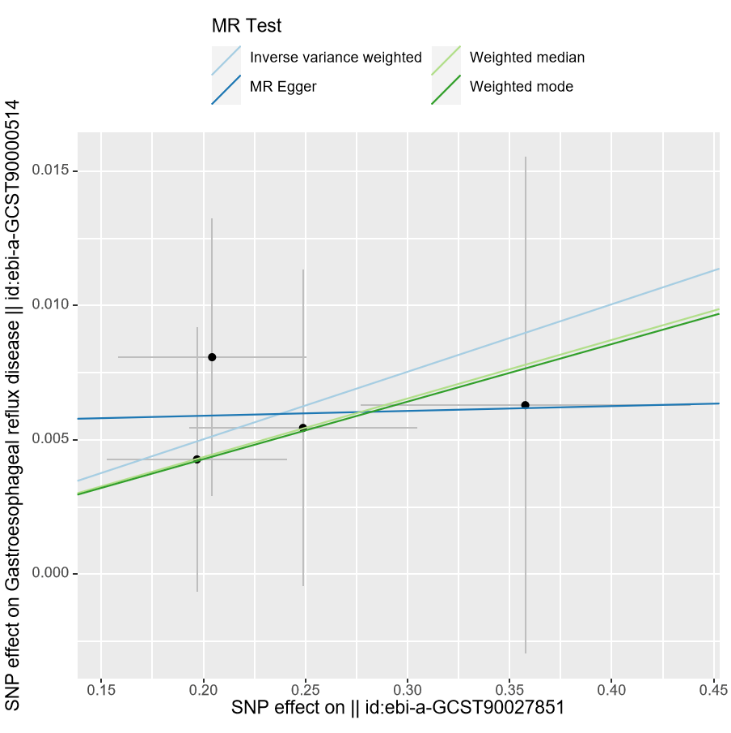


（E）Species *Lachnospiraceae bacterium 5 1 63FAA*

**GERD**

**Supplementary Material 3.** The scatter plots the 4 MR approaches for the association between gut microbiota and FD, IBS and GERD.
